# Burden of disease in Colombian Orinoquia Region, 2017

**DOI:** 10.12688/f1000research.124503.2

**Published:** 2023-11-20

**Authors:** Oscar Gutiérrez-Lesmes, Hugo Grisales-Romero

**Affiliations:** 1 Escuela de Salud Pública, Universidad de los Llanos, Villavicencio, Meta, Colombia; 2 Grupo Demografía y Salud, Universidad de Antioquia, Medellín, Antioquia, Colombia

**Keywords:** Health Status Indicators, Health Profile, Population Health, Global Burden of Disease, Disability-Adjusted Life Years.

## Abstract

**Background:**

Population health diagnoses are a fundamental tool to guide health policies and programs, and consequently, public health requirements. In this perspective, the burden of disease in inhabitants of Colombian Orinoquia is quantified for the first time.

**Methods:**

A descriptive population-based study that was based on secondary sources was carried out, which aimed at measuring the burden of the disease in the Colombian region of Orinoquia, using the simplified synthetic indicator of disability-adjusted life years (DALYs) of the global health estimation methodology. We used mortality records from the National Administrative Department of Statistics (DANE) and service provision records from the Ministry of Health and Social Protection of Colombia, both records from the year 2017, available on the Integrated Social Protection Information System.

**Results:**

288,740.2 DALYs occurred (95% UI 210,714.6-382,948.8), with higher reports for men (59%); group of non-communicable diseases accounted for 62.3% of DALYs (179,993.6, 95% UI, 115,030.2-268,405.0), followed by external cause injuries group which contributed 24.6% (71,000.0, 95% UI, 25,638.1-134,013.1), and group of communicable, maternal, neonatal, and nutritional disorders which contributed 13.1% (37,746.0, 95% UI, 28,048.0-50,239.7). Interpersonal violence was the primary cause specific of DALYs with 9.8% of the burden, (28,290.0, 95% UI, 7,365.1-64,208.1).

**Conclusions:**

Most DALYs in Orinoquia are produced by non-communicable diseases (NCD), largely caused by neoplasms and cardiovascular disease, which increased with age. However, when considered by specific cause of illness or injury, interpersonal violence is indicated as the main cause of DALYs, affecting mainly young men, possibly as an expression of social inequality, substance use, criminality, and insecurity. It is important to highlight that this region has been recognized as an area of armed conflict, drug trafficking, and poverty.

## Introduction

Population health diagnoses are highly relevant since they allow the determination of health requirements and support strategies for policy orientation and health intervention programs. From this perspective, estimating the burden of disease of a population using the synthetic indicator of disability-adjusted life years (DALYs) allows the identification of health status as well as the definition of intervention priorities in this population. In this context, the World Health Organization (WHO) has been measuring the burden of disease for more than two decades and has been using it for population health diagnoses globally, regionally, and nationally in member countries through DALYs aiming to guide public policies and providing diagnostics for the planning of health systems, policies, and programs ( Dessu, Girum, Geremew, and Zeleke, 2020; Fitzmaurice *et al.,* 2019; Hay *et al.,* 2017; Lai, Habicht, and Kiivet, 2009; Melaku *et al.,* 2018; Mokdad *et al.,* 2018; Murray *et al.,* 2012; Nomura *et al.,* 2017; Noordhout *et al.,*2018; Vos *et al.,* 2020; Yoon and Yoon, 2016; Zhou *et al.,* 2019).

DALYs, as a composite indicator, implement specific values to quantify morbidity (not as the frequency of occurrence of the disease but as the time lived in states of less-than-optimal health) and mortality (not as the number of deaths but as the time that an individual lost by dying before reaching life expectancy), unifying them under the same unit of measurement, time (specifically years of life), which allows the comparison between diseases regardless of their etiology, pathogenesis, or symptomatology. DALYs balance the assessment of health events of low lethality and low prevalence with prolonged periods of health impairment *versus* those of high prevalence and/or lethality, solving antagonisms generated by simple indicators of morbidity and mortality in decision-making.

DALYs measure the absence of health through healthy life-years lost in the population of a territory. This time is composed of the sum of years of life lost (YLL) due to premature death and years of life with disability (YLD). YLL are the years that an individual stops living having died before reaching a standard theoretical life expectancy. In this study, 92 years was used as the maximum standard theoretical age for men and women—a value recommended by WHO and corresponding to a projection for 2050 made by the United Nations Population Division ( World Health Organization, 2017). YLD are the years that a person lives in a suboptimal health condition or with health impairments in any of the domains of mobility, self-care, activities of daily living, pain, discomfort, anxiety, depression, social participation, or cognition ( Salomon *et al.,* 2015).

The use of the indicator DALYs in Colombia has been limited. In terms of the number of studies, global burden studies have been carried out with national coverage ( Peñaloza *et al.,* 2014; Rodríguez-García, Peñaloza-Quintero, and Amaya-Lara, 2017) or subnational ( Grisales, *et al.,* 2016), and studies of specific health conditions ( Castañeda-Porras and Segura, 2018; Nieto-López, Grisales-Romero *et al.,* 2022). In Colombian Orinoquia, which comprises the four departments of Arauca, Casanare, Meta, and Vichada, and is inhabited by about 1.7 million people, who are the object of study of this research, there have been no burden of disease studies performed nor measurements of the non-fatal effects of diseases that can be used as a diagnosis of health status to guide health policies and programs taken.

Not having studies that measure the non-fatal effects of the disease causes an underestimation of the impact that these diseases have on the population, affecting decision-making processes. This study allowed for the measurement of the burden of disease that occurred in 2017 in the Orinoquia Colombiana inhabitants for the first time, establishing evidence to prioritize health decision-making.

## Methods

### Ethical statement

This study was classified as minimal risk and it was approved by the Ethics Committee of the National Faculty of Public Health of the University of Antioquia at session 228 of February 21, 2020 (21030002-0038-2020). In addition, requirements of the Gather guide ( Stevens *et al.,* 2016) and the Declaration of Helsinki were applied ( WMA, 2018).

Data are derived from secondary information sources and were collected by state entities with objectives framed in national policies of health care and surveillance. Their collection is regulated by rules that include the mandatory registration of this information by government authorities and data drawn from a public, freely accessible source and stored in electronic format in the Integrated Social Protection Information System (SISPRO, by its acronym in Spanish) warehouse.

### Consent

Consents were non-applicable in this study, since unit of analysis is a conglomerate of data by spatial unit distributed by age groups, sex, and cause, not individuals. Researchers were required to identify or contact any subject to obtain data. In addition, the Council for International Organizations of Medical Sciences (CIOMS) Guideline 12 defines that it is not necessary to obtain informed consent, when data are collected in the context of routine clinical care, are of mandatory registration, and are population-based, as was the case of state databases used in this study.

### Study design

A descriptive, retrospective study of the burden of disease in Colombian Orinoquia was carried out using the indicator DALYs to measure the burden of disease based on the metrics of the WHO Global Health Studies ( World Health Organization, 2017).

### Unit of analysis

Diseases and injuries were considered, according to the frequency of occurrence of the disease or mortality. For the definition of causes of disease or injury, the main diagnosis was used (for morbidity) and the basic cause of death (for mortality). Miscoded mortality cases were included by proportional redistribution. The considered variables were age, illness or injury, sex, and territory. Classification of causes of mortality and morbidity, along with each of the indicators (YLL, YLD, DALYs) was carried out according to the Global Burden of Disease Study (GBD) guide ( Mathers *et al.,* 2001) in a three-level structure with their respective mutually exclusive and exhaustive categories.

### Information sources

The disease and mortality data were obtained from state databases of the Ministry of Health and Social Protection (MSPS, by its acronym in Spanish) of Colombia ( https://www.minsalud.gov.co/Portada2021/index.html), housed in the warehouses of the Integrated Information System of Social Protection (SISPRO), which contains the mortality records of the Non-Fetal Mortality database of the Unique Registry of Affiliates (RUAF, by its acronym in Spanish), and the morbidity cases of the data from the Individual Registry of Health Services Delivery (RIPS, by its acronym in Spanish) database. We accessed the SISPRO server, connecting through a SQL Server Analysis Services cube in Excel, to download mortality data (RUAF) and disease data (RIPS). Using pivot tables, we consolidated disease data ( Gutierrez, 2022a), and mortality data ( Gutierrez, 2022b), from Orinoquia in 2017. This is only able to be accessed using the username and password assigned by the MSPS, under Law 1581 of 2012, letter d, “for historical, statistical or scientific purposes,” ( Ley Estatutaria 1581, 2012).

For demographic information, national population projections from 2017 published by the National Administrative Department of Statistics ( https://www.dane.gov.co/index.php) (DANE, by its acronym in Spanish) were used ( Gutierrez, 2022c).

### Inclusion criteria

All morbidity and mortality cases that occurred in Colombian Orinoquia in 2017 that were registered in the aforementioned information sources, which had an estimate of the disability weight in the table of sequelae, status, and health descriptions and weight of disability that were published in GBD 2019 were included ( Vos *et al.,* 2020).

### Exclusion criteria

Records with undefined cause of death, undefined illness, and fetal death record were not included.

**Data processing**

The data was downloaded from the SISPRO server, where the mortality and morbidity records are housed, reported in the health service provider network and the Institute of Legal Medicine of the Orinoquía region. Tthese reports are made by the treating physician or the forensic doctor through electronic platforms, with the medical reports consolidated in SISPRO, after quality control.

### Bias control

Data extracted from secondary sources are exposed to biases of the information system that collects them. Biases were identified, and actions were performed in the following fashion: First, competing risk biases were identified in the classification of cause of death; in this case, the causes of death were attributed to the basic causes (theoretical assumption) of each of the deaths. The basic cause of death was defined by the treating physician, who prepared the death certificate in one of the cases registered in the Vital Statistics System. Secondly, healthcare access bias was identified; for its control, all mortality cases from health care providers network were involved, including the different forms of participation in the system (with different types of insurance or without insurance), along with the cases reported by the National Institute of Legal Medicine which reports deaths that occurred outside the hospital network. Thirdly, underreporting biases were identified ( Organización Panamericana de la Salud, 2017): completeness of mortality reports in the vital statistics system in Colombian Orinoquia was estimated using the Hill method ( Gutierrez-Lesmes and Grisales-Romero, 2020) subsequently the underreporting was adjusted by expanding the data.

Due to the lack of availability of comparable sources for morbidity, it was impossible to control underreporting of morbidity. Moreover, there was partial control over misclassification bias. According to the methodology Global Health Estimates (GHE), only miscoded cases (ICD-10) were adjusted, relocating them proportionally based on sex and age in the causes or events that occurred ( World Health Organization, 2018).

### Statistical analysis

Once the mortality and morbidity databases had been prepared, YLL, YLD, and DALYs were estimated by using WHO's Global Health Estimates simplified formulas ( World Health Organization, 2017).

The YLL, YLD, and DALYs were calculated through a script in Python 3.0 programming language on Jupyter Notebook platform with the NumPy and Pandas libraries in a publicly available repository ( Gutierrez, 2022d). 95% confidence intervals for these indicators were determined by the Bootstrap technique using the XLSTAT Basic + 2021.2.1 program in 1,000 samples with bias corrections. Rate adjustments were made in EPIDAT 3.1. Applying the aforementioned classification, results allowed the presentation of level one, comprised of three groups (Non-communicable diseases (NCD), communicable, maternal, neonatal, and nutritional diseases (CMNND), and external cause injuries (ECI)), level two with 35 categories (subgroups), and level three with 160 categories of causes for diseases or injuries. The variables were also sorted according to sex and age groups (0-9, 10-24, 25-49, 50-74 and 75 or more years old)

### Determination of the burden of disease

Estimation of YLL

The number of deaths from each basic cause was estimated by age groups and sex. This estimate was adjusted by the under-reporting coefficient ( Gutierrez-Lesmes and Grisales-Romero, 2020). Afterwards, estimation of YLL by cause of death (illness or injury) was performed by using the following factors:

**Equation 1.** Basic formula for YLLs.

$${\mathrm{YLL}}_{c,a,s,t}=D_{c,a,s,t}\;e_{x}^{*}$$

D is the number of deaths due to the cause ( *c*) in the age group ( *a*) *,* in sex ( *s*) *,* and year *t.* $e_{x}^{*}$ is the life expectancy at each age (the weighting factor is derived from the standard life expectancy (SLE) recommended by WHO, based on a 92-year-old SLE).

Estimation of YLD

Point prevalence was estimated for each disease or injury according to main diagnosis reported in the RIPS using the following equation:

**Equation 2:** Basic formula for YLDs.

$${\mathrm{YLD}}_{c,a,s,t}={\mathrm{DW}}_{c}*P_{c,a,s,t}$$

DW is the disability weight, P is the prevalence of the disease or injury ( *c*) *,* in the age group ( *a*) *,* according to sex ( *s*), and year ( *t*), according to GBD 2019 disability weights for each health state.

Estimation of DALYs

Once YLL and YLD were estimated, estimation of DALYs due to illness or injury was carried out using the following equation:

**Equation 3.** Basic formula for DALYs.

$${\text{DALYs}}_{c,a,s,t}={\mathrm{YLD}}_{c,a,s,t}+{\mathrm{YLL}}_{c,a,s,t}$$

( *c*) is disease or injury *,* ( *a*) the age group *,* ( *s*) the sex ( *s*), and ( *t*) is year

## Results

### Cases of illness and death by department and region (by disease group)

The disease and mortality data downloaded from SISPRO, allowed to consolidate the following cases: in the vital statistics system, of the 7,011 cases of death that were registered for 2017, distributed in 1,013 ICD-10 codes, 4,180 occurred in men (59.6%); while in the Health Services Delivery database for 2017, of the 997,159 cases of illness or injury that were registered, distributed in 6,842 ICD-10 codes, 600,784 occurred in women (60.2%). The department of Meta registered the highest number of deaths and illnesses, compared to the other departments of the region, with 63% of cases of death and 51% of morbidity ( Table 1). Regarding disease groups, NCD registered the highest mortality rates and morbidity proportions in the region and its departments ( Table 1).

**Table 1.** Frequencies of death and morbidity by department and region (by disease group).

	Mortality					Morbidity			
		CMNND	NCD	ECI	Total	CMNND	NCD	ECI	Total
	**Department**								
Arauca	Cases	136	711	219	1 066	45 687	93 350	11 734	150 771
	% *	13	67	21	100	30	62	8	100
	% †	16	14	19	15	16	15	15	15
	Rate ‡	54	284	87	426	18 238	37 265	4 684	60 187
	Rate ‡ §	59	344	91	494	18 088	41 523	4 832	64 444
Casanare	Cases	155	917	228	1 300	99 516	188 915	24 962	313 393
	% *	12	71	18	100	32	60	8	100
	% †	18	18	20	19	34	30	32	31
	Rate ‡	38	223	55	316	24 198	45 936	6 069	76 204
	Rate ‡ §	44	310	58	412	24 438	51 888	6 251	82 578
Meta	Cases	533	3 253	643	4 429	135 502	335 457	40 835	511 794
	% *	12	73	15	100	27	66	8	100
	% †	61	65	57	63	47	53	52	51
	Rate ‡	52	318	63	433	13 259	32 825	3 995	50 080
	Rate ‡ §	55	343	63	461	13 361	34 202	4 015	51 579
Vichada	Cases	51	123	42	216	7 815	12 048	1 332	21 195
	% *	24	57	19	100	37	57	6	100
	% †	6	2	4	3	3	2	2	2
	Rate ‡	49	117	40	206	7 438	11 468	1 268	20 174
	Rate ‡ §	54	220	50	324	8 018	15 672	1 557	25 247
	**Region**								
Orinoquia	Cases	875	5 004	1 132	7 011	288 520	629 770	78 869	997 159
	% *	13	71	16	100	29	63	8	100
	Rate ‡	49	280	63	392	16 129	35 207	4 409	55 746
	Rate ‡ §	53	333	65	451	16 228	38 215	4 502	58 947

CMNND: Communicable, maternal, neonatal, and nutritional diseases; NCD: Non communicable diseases; ECI: External cause injuries.

* Disease group percentage in the territory, horizontal reading.

^†^ Departmental percentage by regional total, vertical reading.

^‡^ Numerator: cases by territory and disease group in 2017. Denominator, population projection 2017 by territor. Rate per 100,000 inhabitants.

^§^ Adjusted rate by direct method. Reference population (WHO).

### Fatal effects of diseases and injuries: YLL

These 7,011 cases of death caused a 243,754.4 YLL (95% UI 179,763.8-327.970.2), at a rate of 13,627 (95% UI 10 049.6-18 335.1) AVP per 100 000 inhabitants. Regarding sex, premature death in men exceeded that of women by 63%, and when the loss was compared according to the age group of the deceased, it was highlighted that among young adults from 20 to 29 years of age, men quintupled the death rate of women in the same age range (Underlying data, Table 2 ( Gutierrez (2022d)).

When YLL were segregated by the three large WHO groups, the predominance of NCD was remarkable, followed by ECI and CMNND, respectively. It should be noted that NCD constituted 61% of the total YLL, exhibiting the highest rate of YLL (Underlying data, Table 2 ( Gutierrez (2022d)). A positive gradient was observed in YLL caused by NCD and age without important differences by sex. In contrast, when considering YLL by ECI, the concentration was higher in young men. Concerning CMNND group, a higher number of YLL was observed in children under five years of age, without differences indicated by sex.

In terms of subgroups, respiratory infections, sexually transmitted infections, and nutritional deficiencies accounted for 73% of YLL in CMNND. In these three subgroups, the highest rates corresponded to men, and age-related involvement was in age groups from 0 to 9, 25 to 49, and 50 to 74 years of age, respectively (Underlying data, Table 2 ( Gutierrez (2022d)). Subgroups comprised by cardiovascular disease, neoplasms, and digestive diseases were remarkable for their greater contribution to the burden of premature mortality, with 74% of YLL in NCD. In these first three subgroups, women had a higher rate than men in neoplasms, while men surpassed them in cardiovascular disease and digestive diseases. Regarding age, cardiovascular disease and digestive diseases produced more YLL in the age group of 50 to 74 years of age, while neoplasms were predominant in the 25 to 74 years of age group (Underlying data, Table 2 ( Gutierrez (2022d)). In ECI, violence subgroups (interpersonal violence and self-injuries) contributed to 55% of YLL. In the two groups, men had higher rates than women, and the greatest involvement by age was in the group of 10 to 49 years of age (Underlying data, Table 2 ( Gutierrez (2022d)).

When classified according to YLL rates, the first five subgroups by sex, regardless of disease group (level one), men and women shared three causes: neoplasms, respiratory infections, and cardiovascular disease. Women had higher rates for neoplasms, and in the first five cases, diabetes and birth defects were found. Unlike women, men had higher rates of cardiovascular disease, respiratory infections, and self-injuries; they were also affected by interpersonal violence and road traffic injuries (Underlying data, Table 2 ( Gutierrez (2022d)).

According to the individual classification by diseases or injuries (level three) and their contribution to the total of YLL, the first causes were as follows. In first place, interpersonal violence that produced 12% of YLL in the region was higher in the age group of 25 and 49 years of age, and men accounted for 89% of YLL. In second place, ischemic heart disease (10%), which occurred more frequently in individuals over 50 years of age, was mostly in men (63% of YLL). In third place was road traffic injuries (9%), with greater involvement in the group 10 to 49 years of age, yet 81% of YLL were reported in men. Pneumonia, although with greater involvement in children under nine years of age and in individuals over 50 years of age, 57% of the cases corresponded to men in fourth place. Finally, in fifth place was a stroke in people over 50 years of age, without differences by sex. Other diseases or injuries are shown in in Underlying data, Table 2 ( Gutierrez (2022d). It is important to note that interpersonal violence caused 28,272 YLL, which represented 11.7% of the total YLL in the region.

### Non-fatal effects of diseases and injuries: Years lived with disability (YLD)

The YLDs 9,978,159 cases contributed with a total of 44,985.8 YLD (95% UI, 9,693.6-63,463.7), with a 2,514.9 YLD rate per 100,000 inhabitants (95% UI 1,660-3 547.9). Women lived more years in states of less-than-optimal health, with 56.3% of the total YLD in the region (Underlying data, Table 3 ( Gutierrez (2022e)); most YLD originated in men and women aged 25 to 49 and 50 to 74 years.

According to the large groups, NCD caused the largest disability in the population from Orinoquia with 32,341.1 YLD (95% UI, 19,921.9-47, 461.1), which represents 72% of YLD, with a rate of 1,808 YLD per 100,000 inhabitants (95% UI, 1,113.7-2,653.3); where a higher involvement was found in women with 60% YLD. Regarding age, the main contribution to the YLD was focused mainly between the ages of 25 and 74 years (Underlying data, Table 3 ( Gutierrez (2022e)). When separating the large groups of the classification by sex, the predominance of women was observed in NCD and CMNND groups, while men prevailed in the ECI group (Underlying data, Table 3 ( Gutierrez (2022e)).

When analyzing sub-groups (level two), it was found that three of them were relevant in CMNND with 81% of YLD: enteric infections, respiratory infections, and the subgroup of HIV/AIDS and sexually transmitted infections. Enteric infections are more frequent in women, while HIV/AIDS and sexually transmitted infections are more frequent in men. Concerning age, these three subgroups had a higher concentration of the YLD between 25 and 49 years of age (Underlying data, Table 3 ( Gutierrez (2022e)). Regarding NCD, the first three subgroups, based on their contribution to the total group, were musculoskeletal disorders, neurological disorders, and mental disorders were found. This represented 50.4% of YLD and were more frequent in women. Mental and neurological disorders were more common in the age group of 25 to 49 years, while musculoskeletal disorders were more common in the 50 to 74 years group (Underlying data, Table 3 ( Gutierrez (2022e)). In ECI, unintentional injuries and road traffic injuries were the most relevant subgroups and accounted for 99% of YLD with greater involvement in men aged between 25 and 49 years (Underlying data, Table 3 ( Gutierrez (2022e)).

The ranking of the top five causes or diseases or injuries (level three) was established, these causes generated 42.5% of YLD occurred in the region. In first place was dorsopathies (17,4%) with higher occurrence between 25 and 49 years; in second place, diarrheal diseases (8.5%); and in third place was migraines (5,1%) with greater involvement in the group of 25 to 49 years, followed by gastritis (3.8%) in fourth place. Finally, in fifth place, HIV/AIDS (3.2%) was found. Regarding sex, women were more affected by dorsopathies, migraines, and gastritis, while men were more affected by HIV/AIDS. Concerning age, the cases were concentrated on people aged 25 to 49 years in all diseases, except for diarrheal diseases, which predominated in children under nine years of age (Underlying data, Table 3 ( Gutierrez (2022e)).

### Relationship between YLL and YLD

The contribution of YLL was higher in the burden of disease in Orinoquia. 84.4% of DALYs were due to premature death; in reference to each of the large groups, the contribution of YLL ranged from 77.8% in the CMNND group to 94% in the ECI group; which means that for every year lived with disability by the inhabitants of the region, the loss of 5.4 years of life occurred due to premature death.

The YLL/YLD relationship by disease subgroups showed the predominance of YLL in 17 of the disease subgroups, among which were neoplasms, cardiovascular disease, self-injuries and interpersonal violence, road traffic injuries, respiratory infections and tuberculosis, digestive diseases, unintentional injuries, lower respiratory infections, diabetes, birth defects, chronic respiratory diseases, kidney disease, neonatal disorders, Hemoglobinopathies, maternal disorders, nutritional deficiencies and sexually transmitted infections. YLD predominated in 11 subgroups: substance use disorders, endocrine, metabolic, blood and immune disorders, sense organ diseases, mental disorders, neurological disorders, musculoskeletal disorders, otitis media, oral disorders, gynecological diseases, and enteric infections.

### Fatal and non-fatal effects of the disease (DALYs)

**Figure 1.**
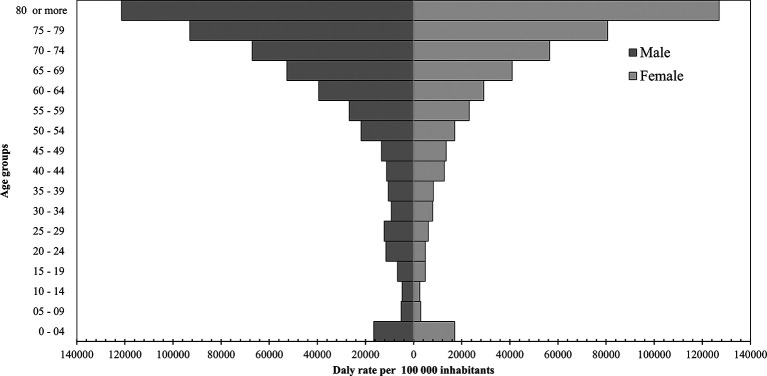
Distribution of disability-adjusted life years (DALYs) by sex and age.

The burden of disease in Orinoquia was estimated at 288,740.2 DALYs (95% UI, 210, 714.6-382,948.8), and a rate of 16,141.9 DALYs per 100,000 inhabitants (95% UI, 11,779.9-21,408.6); 59.1% of DALYs occurred in men, and 60% of them belonged to the age group 25 to 74 years (Underlying data, Table 4 ( Gutierrez (2022f)). The high rate present in children under five years of age is remarkable when compared with age groups of five to 49 years, and it was noticeable that in the age group of 20 to 29 years, the DALYs rate of men doubled that of women ( Figure 1).

Results based on the three large groups showed that NCD produced the greatest burden of disease in Orinoquia, causing 179,993.6 DALYs, (95% UI, 115,030.2-268,405), 62.3% of the health gap in the region, followed by the group of ECI, to which 24.6% of DALYs were attributed. It was highlighted that, for each DALYs per CMNND, approximately 4.7 DALYs per NCD were indicated. Estimates by sex revealed the excess of DALYs in men as the main difference when considering ECI, as they accounted for 81.9% of DALYs (Underlying data, Table 4 ( Gutierrez (2022f)).

**Figure 2.**
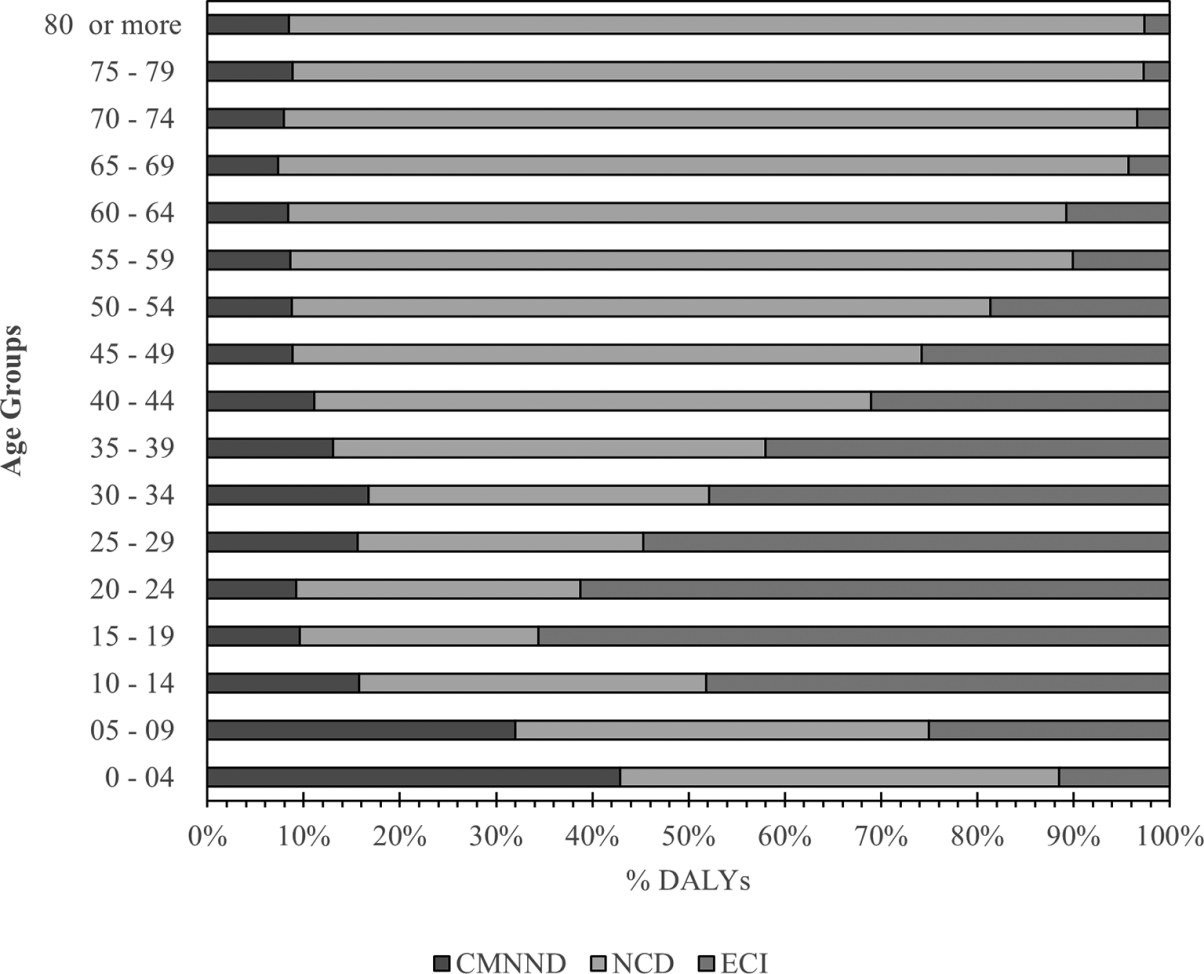
Distribution of large disease groups by age groups. Notes: NCD: Noncommunicable diseases; ECI: External cause injuries; CMNND: Communicable, maternal, neonatal, and nutritional diseases. DALYs: Disability-adjusted life years.

Concerning age, a positive gradient was observed between age and DALYs due to NCD (from the age of 10 years); for CMNND, the greatest burden occurred in individuals under 10 years of age, causing 40.8% of DALYs in that age group. DALYs due to ECI predominantly affected people aged between 10 and 49 years, causing 60.5% of DALYs in the group ( Figure 2).

Regarding subgroups (level two), respiratory infections (including TB), sexually transmitted infections (including HIV/AIDS), and nutritional deficiencies caused 57.1% of DALYs in CMNND. In these subgroups, men accounted for most DALYs. Concerning age, the greatest accumulation of DALYs was due to respiratory infections in the 50-to-74-year-old group, followed by sexually transmitted infections, in the 45 to 49 years group, and nutritional deficiencies in individuals under 10 years of age. On the other hand, neoplasms, cardiovascular diseases, digestive diseases, and diabetes produced 64.5% of DALYs in NCD. With regard to sex, women were the largest contributors to DALYs due to neoplasms, while among men, cardiovascular diseases and digestive diseases were predominant. In terms of age, neoplasms mostly affected people aged 25 to 74 years, whereas heart diseases and digestive diseases had the greatest effect on people between 50 and 74 years. Finally, the self-injuries, interpersonal violence, and road traffic injuries subgroups caused 83% of DALYs, mainly in men aged 25 to 49 years (Underlying data, Table 4 ( Gutierrez (2022f)).

In the analysis of subgroups by sex, the five highest rates of DALYs showed that men and women shared neoplasms and cardiovascular disease as first causes. However, the difference by sex is remarkable. While personal violence and self-injury predominated in men (in that order), road traffic and unintentional injuries did so in women. The first cause of DALYs was neoplasms, and in their first five causes, digestive diseases, work-related musculoskeletal disorders and respiratory infections were found (Underlying data, Table 4 ( Gutierrez (2022f)).

**Figure 3.**
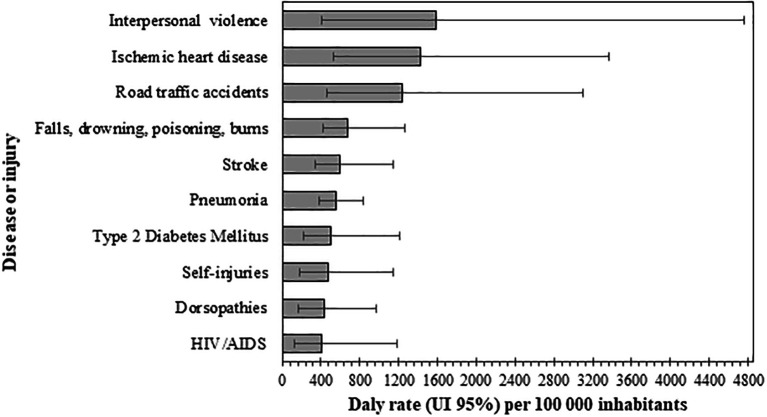
Disability-adjusted life years (DALYs) rates of the first 10 diseases or injuries, Orinoquia 2017.

Respecting diseases and injuries (level three), the ten leading causes accounted for 49% of DALYs in the region (Underlying data, Table 4 ( Gutierrez (2022f)). According to their rates, the first three diseases or injuries that contributed to burden were the following: interpersonal violence 1,581.5 DALYs per 100,000 inhabitants (95% UI, 411.7-3,589.5), ischemic heart disease 1,430.4 DALYs per 100,000 inhabitants (95% UI, 528.9-2,471.3), and road traffic injuries 1,235.8 DALYs per 100,000 inhabitants (95% UI, 459-2,323.9), as can be seen in Figure 3.

**Figure 4.**
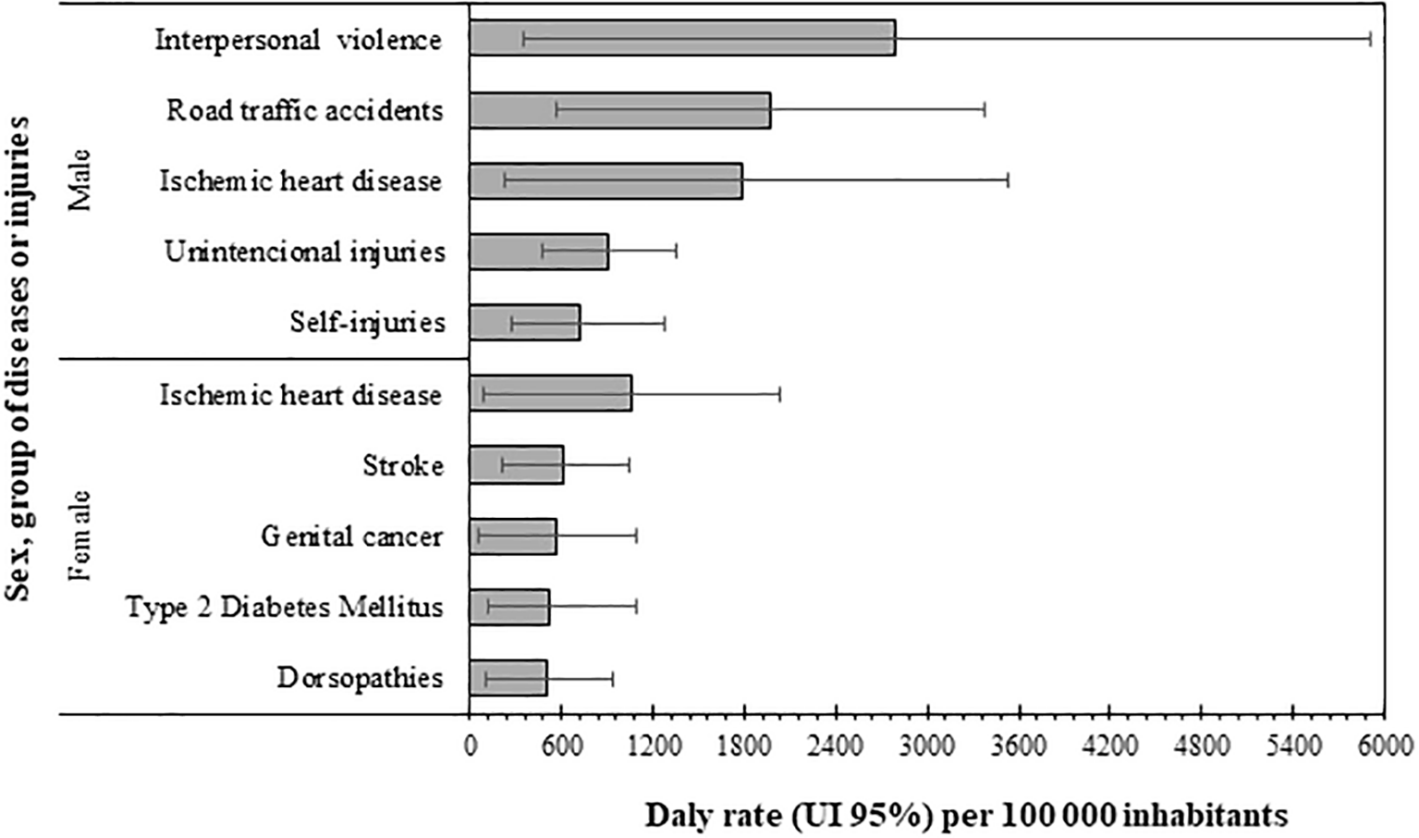
Disability-adjusted life years (DALY) rates of the first five diseases or injuries by sex, Orinoquia 2017.

However, when analyzing the first five causes of DALYs by sex, important differences between men and women were found ( Figure 4). In women, the following group (all belonging to the NCD) predominated (in descending order): ischemic heart disease, stroke, genital cancer, type 2 diabetes mellitus, and dorsopathies. Conversely for men, the first five causes were the following: interpersonal violence, road traffic injuries, ischemic heart disease, unintentional injuries, and self-injuries. Four of these belong to the ECI group, and a prominent contribution of interpersonal violence was found. The only disease present in both sexes in its first five causes was ischemic heart disease.

Concerning the most affected age groups in each of the ten global causes, interpersonal violence and road traffic injuries mainly affected people aged between 15 and 34 years; unintentional injuries were common in all ages with a slight increase in individuals over 75 years of age; HIV/AIDS affected people aged between 20 and 44 years; and dorsopathies impact individuals from 14 years. Type 2 diabetes mellitus, ischemic heart disease, and stroke were present after 60 years of age, and pneumonia involved people aged between zero and four years and over 69 years of age (Underlying data, Table 4 ( Gutierrez (2022f)).

## Discussion

This study showed the experience of the first measurement of the burden of disease in Colombian Orinoquia. It required the use of secondary data sources, whose custody is the responsibility of the national health authority. Morbidity and death data were validated according to the known strategies for this purpose and based on validation and data cleaning protocols. Although this study did not aim to explain determining factors of health in Orinoquia, analyzed diseases and injuries have already been related to lifestyle, biological, environmental, and sociocultural factors as well as inequalities ( Roth *et al.,* 2018; Sánchez-Ledesma *et al.,* 2018; Vos *et al.*, 2020).

It is important to note that the center of the discussion is the burden of disease, which unifies the time lost by premature death and the time in states of less-than-optimal health (disability) in an indicator without separating the relationship of the natural history of each disease, which means not observing disability or premature death caused by disease or injury as separate events. According to this approach, groups and subgroups of disease and/or injury and diseases or injuries that produce the greatest burden of disease in Orinoquia and its departments are presented.

When compared to ECI and CMNND, NCD provided the highest burden of disease -the major cause of loss of health, in terms of DALYs (YLL, YLD)- affecting both men and women substantially from 50 years of age. A similar finding was made in reports from Colombia, Latin America, and other parts of the world ( Dantés-Gómez *et al.,* 2011; Grisales *et al.,* 2016; Peñaloza *et al.,* 2014) and estimates in Uruguay ( Ministerio de Salud, 2010), Argentina ( Borruel, Mas, and Borruel, 2010), Chile ( Universidad Católica de Chile, 2008), Mexico, Brazil, Costa Rica and Peru ( Dantés-Gómez *et al.,* 2011). Worldwide studies have reported similar results ( Hay *et al.,* 2017; Kyu *et al.,* 2018; Nomura *et al.,* 2017; Noordhout *et al.,* 2018; Vos *et al.,* 2020).

When disaggregating the burden from NCD, based on YLL and YLD in Orinoquia, it was defined that premature mortality (YLL) played a fundamental role, accounting for 77.9% of DALYs by NCD in the region, similar to what was found in Colombia ( Grisales *et al.,* 2016; Peñaloza *et al.,* 2014), other countries of America ( Borruel *et al.,* 2010; Dantés-Gómez *et al.,* 2011; Ministerio de Salud, 2010; Universidad Católica de Chile, 2008), and other parts of the world ( Hay *et al.,* 2017; Kyu *et al.,* 2018; Lang *et al.,* 2018; Melaku *et al.,* 2018; Noordhout *et al.,* 2018). Regarding years lived with disability (YLD) produced by NCD in Orinoquia, these were 2.8 times higher than those produced by CMNND and 6.5 times more years than those caused by the ECI.

Regarding YLD – suboptimal health status – due to non-fatal effects of disease, NCD were also consolidated as the group with the greatest effects on the health gap in Orinoquia, with predominance in women. These results did not differ from studies in the country, such as those carried out in Medellín for septennium 2006-2012 ( Grisales *et al.,* 2016), for Colombia, national estimations for 2005 and 2010 ( Acosta-Ramírez, Peñaloza, and Rodríguez-García, 2008 and Peñaloza *et al.,* 2014) and Latin America and the Caribbean, Institute for Health Metrics and Evaluation (IHME) for 2017 ( IHME, 2021a).

The superiority of NCD in the contribution to the burden of disease (DALYs) by premature deaths (YLL) and by the generation of disability (YLD) has been explained as an effect of population aging and the world’s economic and industrial development ( Acosta, 2013), which has changed population profiles of the health gap, moving from a disease burden that was mainly caused by communicable diseases to population profiles where the health gap is mainly generated by NCD ( Hay *et al.,* 2017; Lang *et al.,* 2018; Rodríguez-García *et al.*, 2017; Rodríguez-Vargas, Martínez-Almanza, Pría-Barros, and Menéndez-Jiménez, 2004; Vos et al., 2020). Population aging is a demographic and social condition defined by the decrease in the birth rate and the increase in elderly people due to the increase in life expectancy ( Ochoa-Vázquez, *et al.,* 2019; Ramos *et al.,* 2014). In terms of the burden of disease, this behavior has been presented as the first cause for the world from 2010 to 2019 ( Hay *et al.,* 2017; James *et al.,* 2018; Murray *et al.*, 2012; Vos *et al.,* 2020).

The predominance of NCD in premature mortality (YLL) and disability (YLD) as a cause of disease burden is not uncommon and is part of the world’s health profile in the 21 ^st^ century because its multiple causes are being amplified. Such causes include socioeconomic development, urbanization, industrialization, lifestyle change, control over infectious diseases, and increased life expectancy along with recognized risk factors, such as substance use, obesity, hypertension and sedentary lifestyle ( Acosta, 2013; Martínez, 2016; Ramos *et al.,* 2014). In addition to the aforementioned, clinical characteristics of chronicity and the defects of health systems are added ( Landrove-Rodríguez *et al.,* 2018).

For Orinoquia, DALYs due to NCD increased by age from 50 years of age. This behavior was recurrent for Colombia in 2017 ( IHME, 2021b), Latin America ( IHME, 2021a; Vos *et al.*, 2020), and other parts of the world ( James *et al.*, 2018; Kyu *et al.,* 2018; Vos *et al.,* 2020). The fact that the age groups that are most affected by NCD are those over 50 years of age has been linked to the natural physiological deterioration and the accumulation of exposure to NCD triggers ( Martínez, 2016). This involvement was also observed in the group of individuals under 50 years of age, specifically in women. This is due to the increase in neoplasms at an early age ( Fitzmaurice *et al.,* 2017; Global Burden of Disease Cancer Collaboration, 2018; Vos *et al.,* 2020); additionally, disparities by sex have been identified as a product of differences in risk exposure, occupational profiles and specific screening programs ( Martínez, 2016; Pardo and Cendales, 2018; Salas Zapata and Grisales Romero, 2010).

Regarding subgroups of disease that constitute NCD, neoplasms and cardiovascular diseases were highlighted for their contribution to premature mortality along with musculoskeletal disorders and neurological disorders for their contribution to days lived with a suboptimal state of health. In neoplasms and cardiovascular disease subgroups, more than 95% of DALYs were originated from premature mortality (YLL); additionally, neoplasms in Orinoquia affected mainly women aged 25 to 74 years. Predominance in women in this age group was similar to findings described in Colombian studies performed in the departments of Nariño ( Rocha-Buelvas *et al.,* 2014), Santander ( Esquiaqui-Felipe *et al.,* 2012), and Medellin ( Grisales *et al.,* 2016). Predominance of neoplasms in women at an early age could be explained by the burden generated by breast and cervical cancer as these represented the main types of cancer in women in Orinoquia, who have access to screening programs for early detection, which allows greater detection of these illnesses at young ages ( Díaz, Piñeros, and Sánchez, 2005; Murillo *et al.,* 2009; Pardo and Cendales, 2018; Salas Zapata and Grisales Romero, 2010).

The health gap caused by neoplasms is similar to that found in Colombia for measurements made by the IHME for 2017 and 2019 ( IHME, 2021b; Vos *et al.,* 2020) and in the study carried out in Medellin ( Grisales *et al.,* 2016). Besides, it is comparable to the world’s situation, where neoplasms occupied the first causes of DALYs and YLL worldwide ( Fitzmaurice *et al.,* 2017, 2019; Vos *et al.*, 2020). It is important to discuss that the similarity in the contribution of neoplasms to the health gap between the territories mentioned is part of a global trend. The burden originated by the neoplasms have increased worldwide since 1990 and are expected to continue in the process of epidemiological transition due to aging and exposure to carcinogenic factors ( Fitzmaurice *et al.*, 2017). Therefore, neoplasms are recognized as a threat to Colombian and human development ( Ospina, *et al.,* 2015). The fact that the majority of DALYs due to neoplasms In Orinoquia are caused by premature death is a common situation in the world, as reported in a systematic analysis of DALYs due to neoplasms carried out for 195 countries by factors related to detection and care ( Global Burden of Disease Cancer Collaboration, 2018).

According to the contribution to total DALYs, the subgroup for cardiovascular diseases came in first place in Orinoquia, especially in people over 50 years of age and with predominance in men, which was similar to findings in related studies in Medellín ( Grisales *et al.,* 2016), Colombia, Latin America, and other parts of the world ( Peñaloza *et al.,* 2014; Dantés-Gómez *et al.,* 2011; Vos *et al.,* 2020). The particular similarity in the great gap caused by cardiovascular diseases (DALYs) and in premature mortality (YLL) denotes unique global causes in populations where disease burden studies have been carried out ( Cuadras and Rovira, 2014; Hay *et al.,* 2017; James *et al.,* 2018; Lang *et al.,* 2018; Vos *et al.*, 2020). Cardiovascular disease is not only attributed to population growth and aging ( Rodríguez, Machado, and Luna, 2018) but also to biological, environmental, and socioeconomic factors that have an influence on lifestyles that promote risk factors, such as alcohol and tobacco use and high-calorie foods. In addition, inequalities and cultural differences that hinder access to health services and healthy foods and generate physical inactivity, which leads to obesity ( Fernández-González and Figuerola, 2018; García, 2018; Gómez, 2011; González, *et al.,* 2020; Mejía-Merino and Arango-Álzate, 2012), also increase the odds for cardiovascular disease. The aforementioned emphasizes the need for regional policies to intervene a problem that it is expected to continue to increase ( Acosta, 2013; Fortich and Gutiérrez, 2011).

Ischemic heart diseases, which are from the subgroup of cardiovascular diseases, had an important contribution to the burden of disease in the study period, taking second place in Orinoquia; which was similar to findings reported by IHME for Colombia, where ischemic heart disease was the second leading cause of DALYs in 2017 and in 2019 ( IHME, 2021b; IHME, 2021). It was comparable to findings reported for Latin America and the Caribbean, where it was the first leading cause ( IHME, 2021a), and for the world, as the second cause of DALYs in 2019 ( Vos *et al.,* 2020). Concerning premature mortality, YLL, ischemic heart diseases were the second cause of YLL in the world in 2010 and the first in 2019, especially from 50 years of age ( Murray *et al.,* 2012; Vos *et al.,* 2020). Similar behavior was found in Orinoquia with predominance in men and in group ages from 50 years of age, and it was found in Colombia as a part of the NCD group. This behavior and the similarity between territories like Colombia (which includes the Orinoquia), Latin America, and other parts of the world can be explained by the presence of common factors in all territories such as aging, lifestyles, and environmental factors ( Albala, 2020; Belasco and Okuno, 2019; Cardona and Peláez, 2012; Nieto and Alonso, 2007; Rivillas, *et al.,* 2017; Rodríguez *et al.,* 2018). In addition, for ischemic disease, eating habits are described ( Ballesteros-Vásquez, *et al.,* 2012; Cabezas-Zábala, Hernández-Torres, and Vargas-Zárate, 2016). The consumption of substances such as alcohol, tobacco, and sodium along with sedentary lifestyles and obesity are mentioned as the main promoters of morbidity and mortality from ischemic heart disease ( Acosta, 2013; Evans-Meza, Pérez-Fallas, & Bonilla-Carrión, 2019; Núñez-González, *et al.,* 2018), which translates into the burden of disease described.

It is important to note that results reported by Peñaloza et al ( Peñaloza *et al.*, 2014) for Colombia are dissimilar to the findings mentioned for Orinoquia and its departments. In that study, ischemic heart diseases occupied the thirteenth position in the cause of DALYs. This difference may be related to the metric applied by Peñaloza, who applied discount rates on the burden based on age and a lower life expectancy, which causes a much greater underestimation of the burden in advanced diseases, as is the case of ischemic heart disease.

Regarding disability in NCD, two subgroups were remarkable in the region: musculoskeletal and neurological disorders. This was similar to what was found for Colombia in 2017 and 2019 ( IHME, 2021), in Latin America ( IHME, 2021a), other countries in the world in global burden measurements for 2010 and 2019 ( Murray *et al.,* 2012; Vos *et al.,* 2020). The large volume of disability originated by these subgroups is related to their chronicity, low mortality, high prevalence, and involvement in activities of daily life of the people affected ( Martínez-Plaza, 2009; Ramírez-Perdomo, Salazar-Parra, and Perdomo-Romero, 2017).

Musculoskeletal disorders are one of the most important problems in human occupational health worldwide due to sequelae and chronic pain without mortality, hence its great impact on the generation of disability ( Martínez-Plaza, 2009). In this subgroup, dorsopathies were in the first place in Orinoquia, especially among people aged between 25 and 49 years, a situation comparable to that of Colombia, Latin America, and other parts of the world ( IHME, 2021a; IHME, 2021; Murray *et al.,* 2012; Vos *et al.,* 2020). These disorders have been classified as an occupational health problem that occurs at productive ages but has also become frequent in the general population in unpaid activities ( Barreda-Castillo and Bazan, 2019; Casado-Morales, Moix-Queraltó, and Vidal-Fernández, 2008), becoming a disability problem ( Carvalho-Rodrigues and da Costa, 2018). This similarity in musculoskeletal disorders and dorsopathies in Orinoquia, Colombia and the world is due to the presence of common factors that facilitate the occurrence of these disorders, such as physical (strength, repetition, and postures) and psychosocial factors such as high workload ( Arenas-Ortiz and Cantú-Gómez, 2013; Casado-Morales *et al.,* 2008; Chaves-García, 2014; Márquez-Gómez, 2015). These factors are described as common in Colombia, Latin America, and other parts of the world ( Arenas-Ortiz and Cantú-Gómez, 2013; Bautista-Sánchez and Jímenez-Santiago, 2014; Oliveira, *et al.,* 2021).

Among neurological disorders, migraines produced the greatest disability, especially in the population aged between 10 and 49 years, similar to findings for in Colombia, Latin America, and other parts of the world ( IHME, 2021a, 2021b; Vos *et al.,* 2020). High levels of disability come from its chronicity, impairment in the abilities to carry out activities of daily living in affected individuals, and a high demand for health and care services ( Ramírez-Perdomo *et al.,* 2017; Sáez Ruiz, 2017). Concerning migraines and their occurrence, factors such as obesity and a sedentary lifestyle, smoking, and alcohol consumption are mentioned ( Le *et al.,* 2011; Molarius, Tegelberg, and Öhrvik, 2008; Peterlin *et al.,* 2013; Winter *et al.,* 2011), factors that are common for all territories.

ECI accounted for 25% of the burden and were positioned as the second cause of DALYs in the Orinoquia. The important contribution of ECI to DALYs is mainly caused by premature mortality in the young population, corresponding to 82% of DALYs. ( Bejarano Castro, *et al.,* 2006), highlighting their lethality, hence the importance of prevention. The position of ECI is similar to findings reported in DALYs measurements for Colombia by IHME in 2017 and 2019 ( IHME, 2021b; Vos *et al.,* 2020). Comparable results were obtained in Medellín ( Grisales *et al.,* 2016); however, it is relevant to point out differences between the proportions reached. In Orinoquia, they caused 24% of DALYs; according to the IHME report for Colombia in 2017, ECI caused 18.48%, which is superior to results reported in Medellín in the department of Antioquia, where the report was 5.9%. Another Colombian territory with measurements of the burden of disease originated by ECI is Bucaramanga, in the department of Santander, with a DALYs rate caused by ECI for 2017 of 1,200 DALYs per 100,000 inhabitants ( Casadiegos-Patiño, Esquiaqui-Felipe, and Serrano-Diaz, 2021), which is exceeded more than 3 times by the rate calculated for Orinoquia in this study.

The differences found in this study and compared to Medellín and Bucaramanga could be related to the metrics used in these studies, which were different from the ones used in this study, with the consequent underestimation of YLL in Medellín and Bucaramanga, main source of DALYs by ECI ( Rodríguez-García *et al.,* 2017); therefore, it is necessary to be measured with the magnitudes of the estimates by the different metric approaches used although the pattern is consistent.

To contrast these findings, it is very important to specify the two causal pathways that include these ECI subgroups of injuries, which are intentional injuries, are related to violence (self-injuries and interpersonal violence), and caused the most YLL and unintentional injuries that are related to incidents (road traffic and unintentional injuries) ( Castañeda-Porras and Segura, 2018; Concha-Eastman and Clavel-Arcas, 2008) and caused more YLD. These two pathways denote a great difference to understand the behavior of ECI due to their origin and lethality.

Findings on the greater number of YLL occurred in intentional ECI evidenced the greater lethality due to the severity of the injury over unintentional injuries ( Bejarano Castro *et al.,* 2006) and a predominance in men aged between 10 and 49 years, results that are similar to studies in Colombia ( Acosta-Ramírez *et al.,* 2008; Casadiegos-Patiño *et al.,* 2021; Grisales *et al.*, 2016; IHME, 2021), Latin American countries such as Brazil, Mexico, Peru, Chile, Uruguay ( Borruel *et al.,* 2010; Ministerio de Salud, 2010; Universidad Católica de Chile, 2008; Velásquez *et al.,* 2009), and other countries of the world ( James *et al.,* 2018; Kyu *et al., 2018*; Roth *et al.,* 2018; Vos *et al.,* 2020). Disease groups’ behavior by age is consistent with the natural history of the disease. The occurrence of ECI has been related to cultural patterns such as the consumption of psychoactive substances (both illegal and legal, such as alcohol) and masculinity, which, along with weapon carrying, are generators of violence ( Bejarano Castro *et al.,* 2006; Granados, 2017; Ramírez, 2006).

In unintentional ECI (subgroup of unintentional injuries and road traffic injuries), the age range of occurrence was extended to between 10 and 74 years of age. Although the predominance of men persisted, the proportion of women increased almost twice compared to the proportion reached by women in intentional ECI. Changes in premature mortality and the affected population group could be explained by the nature of the injuries that caused it. These included falls, burns, poisoning, drowning, and blows ( Vos *et al.,* 2020), which do not cause premature mortality due to their severity but do cause non-fatal effects (disability). This behavior is derived from aspects described for Colombia, such as age, lifestyle, and work conditions ( Timarán-Pereira, Calderón-Romero, and Hidalgo-Troya, 2017).

Due to differences among available measurements, more clarity is required to compare results originated from this study to Colombia’s profile. According to IHME, the proportion of ECI in 2005 and 2010 for Colombia was 22.79% and 21.7% respectively ( IHME, 2021b), placing ECI as second group causing DALYs. On the other hand, reports of national studies on the percentage of total DALYs caused by ECI in Colombia were 9% in 2005 ( Acosta-Ramírez *et al.,* 2008) and 8% in 2010 ( Peñaloza *et al.,* 2014), placing ECI as the third cause of DALYs. It is essential to highlight that such comparisons between this research on Orinoquia and studies carried out in Colombia in 2005 and 2010 should be made with discretion since the latter used 1993 metrics, which is affected by the underestimation of indicators. In this context, in the last study published by IHME in 2019, a similar metric to that used in this study was applied; and results on ECI are comparable, placing ECI as the second cause of DALYs and YLL ( Vos *et al.*, 2020). Regarding ECI worldwide, this group was established as the third cause of DALYs and YLL ( Hay *et al.,* 2017; Kyu *et al.,* 2018; Mboi *et al.,* 2018; Noordhout *et al.,* 2018; Vos *et al.,* 2016, 2020).

Among ECI, at Level 3, two types of injuries stood out in the Orinoquia due to their magnitude on DALYs and YLL; first, interpersonal violence, followed by road traffic injuries, which is a usual behavior described for Colombia, for Latin America, and for other parts of the world ( Murray *et al.,* 2012; Porras Cataño and Grisales-Romero, 2021; Vos *et al.*, 2020). Interpersonal violence, manifested as homicide or attempted homicide, is the first cause of DALYs, a situation that comes from a violent society as has been described for Colombia ( Bejarano Castro *et al.,* 2006; Marquina, 2017) either due to daily conflicts or political causes ( Martínez, 2016). Other authors propose that it arises from the presence of a masculine identity (rivalry, competition, and justification of superiority over other men) that tends to solve problems, discrepancies, and disagreements through violence ( Cano and Rojido, 2017; Ramírez, 2006), alcohol consumption, and weapon carrying ( Bejarano Castro *et al.,* 2006). Other explanations include precarious jobs and unemployment, leading to criminality and gender roles with a dominant man in a context of violence ( Dávila-Cervantes and Pardo-Montaño, 2018; Granados, 2017; Vazsonyi, Clifford-Wittekind, Belliston, and Van Loh, 2019). These findings are consistent with other studies for 2017 and 2019, where interpersonal violence was placed as the first cause of YLL in Colombia ( IHME, 2021b; Vos *et al.,* 2020). However, it is important to mention that the prevalence of interpersonal violence found in this study is a serious phenomenon, which contrasts with world figures which showed that it was far from being part of the top two causes of YLL in 2010 and 2019. Interpersonal violence barely becomes the 18 ^th^ cause of YLL in the world, 26 ^th^ of DALYs, and 42 ^nd^ of YLD ( Murray *et al.,* 2012; Vos *et al.,* 2020).

Road traffic injuries that took second place in Orinoquia had a behavior that is similar to that of the country ( IHME, 2021b) but that contrast with the position they reached in the world between thirteenth place in 2010 and fourteen in 2019 ( Murray *et al.,* 2012; Vos *et al.,* 2020). The fundamental factors for safe driving recognized are the person, the vehicle, the road, and traffic rules; but the main variable causing traffic incidents is the person. For Colombia, risk behaviors have been described as road traffic injury generators: crossing without looking, speeding, driving under the influence of alcohol, driving on the wrong side of the road, failure to obey traffic signals, among others ( Bejarano Castro *et al.,* 2006). There is also a biological factor related to the reproductive stage to take into account, where individuals take risks and execute risk behaviors such as the aforementioned ( Norza-Céspedes *et al.,* 2014).

CMNND were the group of diseases that caused the least burden in the Orinoquia, a situation that is similar for Colombia, Latin America, and the Caribbean, but not in the global burden, where CMNND placed second. When analyzing the position of CMNND in the countries of the world using classifications that measure development or wealth such as the Socio-Demographic Index ^1^ used by IHME or income levels defined by the World Bank, it is possible to observe a gradient in the proportion of CMNND. These decrease with an increase in the development or the wealth of a country, thus, changes of position in the classification. For instance: CMNND originate 58.8% of DALYs in low-income countries, being the first cause of DALYs; in lower-middle income countries, they produced 35% and were placed in second position as causes of DALYs. On the other hand, in upper-middle income and high-income countries, they produced 9.9% and 4.6% respectively, and CMNND are being located as the third cause ( IHME, 2021d). It is concluded that findings from different studies allow us to note that economic factors may influence the contribution of CMNND to total DALYs ( Berlinguer, 2007; Méndez, 2018; Ortega, 2019; Vargas, 2019), and this would explain our findings.

Among CMNND, respiratory infections, sexually transmitted infections (STI), and enteric infections subgroups were remarkable by their contribution to DALYs (YLL, YLD). These findings are consistent with the results found for Colombia and other parts of the world, since these subgroups are part of the first cases within CMNND ( Dantés-Gómez *et al.,* 2011; Murray *et al.,* 2012; Peñaloza *et al.,* 2014; Porras Cataño and Grisales-Romero, 2021; Vos *et al.,* 2020). Enteric infections are recognized as one of the main causes of morbidity and mortality in developing countries ( Cáceres, *et al.,* 2005), where poverty and sanitation problems (drinking water and sewerage) contribute significantly to the occurrence and greater involvement of early childhood ( Mariños-Anticona, *et al.*, 2014). In Colombia, multidimensional poverty index (MPI) is applied, which measures access to sanitation, drinking water and sewerage, and access barriers for early childhood to health care and services ( Moreno-Gómez, Duarte-Gómez, and Barrientos-Gutiérrez, 2017; Salazar, Cuervo, and Pinzón, 2011). On this scale, Orinoquia has MPI scores ranging from 19.1% to 63.3% in its different departments ( DANE, 2019), which could be accounting for the figures achieved in Orinoquia in 2018.

Finally, findings on the superiority of the fatal effects of YLL compared to the non-fatal effects of YLD component in their contributions to the burden of disease are similar to measurements of the global burden estimated by IHME for Colombia and other parts of the world, with a greater contribution of YLL to DALYs ( Hay *et al.*, 2017; James *et al.,* 2018; Kyu *et al.,* 2018; Vos *et al.,* 2017, 2020), highlighting that premature mortality, which comes from diseases or injuries with high lethality or deficiencies in care of health systems ( Cainzos-Achirica and Bilal, 2021; Dumoy, 2013; Laroze, 2018), is the main source of the health gap of humanity due to the volume of years it produces.

When analyzing by sex, differences between YLL and YLD became evident in Orinoquia, as well as in the rest of Colombia and other parts of the world. Women live longer in suboptimal states of health (i.e., YLD), while men die more and at an earlier age than women (i.e., YLL; Dantés-Gómez *et al.*, 2011; Grisales *et al.,* 2016; Hay *et al.,* 2017; IHME, 2021c; James *et al.,* 2018; Lang *et al.,* 2018; Peñaloza *et al.,* 2014; Vos *et al.,* 2020). This result is expected due to reasons exposed in previous paragraphs which demonstrate that men are more affected by diseases and injuries with greater lethality (interpersonal violence, self-injuries, and road traffic injuries), while women are more affected by non-communicable diseases that produce a greater disability ( Dantés-Gómez *et al.,* 2011; Grisales *et al.,* 2016; Hay *et al.,* 2017; IHME, 2021c; James *et al.,* 2018; Lang *et al.,* 2018; Peñaloza *et al.,* 2014; Vos *et al.,* 2020).

It is important to note that in studies carried out in Colombia, the proportion of DALYs that derived from YLL is lower if compared to what was found in this study, where YLL contribute 84%. In 2010, YLL represented only 21.6% of the burden in Colombia ( Peñaloza *et al.,* 2014), and in Medellín for the septennium of 2006-2012, they only represented 13% ( Grisales *et al.,* 2016). These differences could be explained by the use of the GBD 1993 metric in these studies with a standard life expectancy of 82.5 years for women and 80 years for men and discount rates affecting weighting factors (WF) used to estimate YLL. WF, when using 1993 metric, was from 33.13 and 33.01 for women and men, respectively, at 0 years of age, up to 0.57 and 0.52 to 100 years of age, while in this study, without discount rates and a standard life expectancy of 92 years, a WF of 90.1 in the group of 0-4 years and 1.94 in the group of 100-104 years was produced. Therefore, this difference in the metrics would be responsible for the discrepancies ( World Health Organization, 2013, 2017), as what happened in the measurement of global YLL for 2011. It is estimated that it caused an underestimation of 62% of YLL, causing changes in the relative contribution to YLL and DALYs.

### Limitations

It is important to report limitations, first concerning the quality of data. In terms of reliability, even when data was obtained from the Ministry of Health and Social Protection, non-fetal mortality from RUAF database and health care records from RIPS are considered reliable by quality assurance systems and the strategies applied by authors for bias control, such as misclassification and underestimation of cases. It was not possible to adjust possible errors in the main diagnoses of morbidity or to adjust to underreporting due to the lack of a comparable source of information for morbidity for the execution of available methods.

Methodological limitations were also presented. For example, it was not possible to identify level four categories of the GBD methodology in the ICD-10 records from the state morbidity database and their disability weights, such as applying pain gradients, severity of diagnoses, or identifying the vehicle in road accidents. In the results, it was not possible to adjust YLD by comorbidities, as the morbidity database does not allow the individual identification of cases, since they are grouped by disease or injury in dynamic tables, making available techniques inapplicable. Finally, limitations were presented when comparing and discussing results due to the fact that studies that were carried out in Latin America and Colombia used a metric from the DALYs in the 90s.

## Conclusions

The greatest loss of DALYs in Orinoquia comes from NCD, mainly by neoplasms and cardiovascular disease, which increase with age. The demographic transition for the territory is evident by the greater involvement caused by NCD. Although when grouping causes (diseases and injuries) of DALYs, NCDs are the main contributors to the burden of disease of the territory, when discriminating by specific cause of illness or injury, interpersonal violence is constituted as the main cause of DALYs in Orinoquia, principally affecting young men.

Furthermore, it is important to highlight differences found by sex and age that should lead to policies and programs with differential approaches; the burden of disease in men comes mainly from premature mortality (YLL) due to ECI, while in women, the burden comes from NCD by disability (YLD). Based on life cycles, differences in the causes of DALYs were also found, such as greater involvement by CMNND in children, ECI in adolescents and young people, and NCD in adults and the elderly.

Now, it is important to emphasize that in DALYs that occurred in Orinoquia, YLL surpassed by a great difference YLD in the burden of disease, highlighting that premature mortality is a problem that requires priority and immediate attention in these territories.

## Data Availability

The data on morbidity and mortality used in this study were obtained from a third party, the Ministry of Health and Social Protection of Colombia (MSPS). To download them, a username must be requested to MSPS. Requests for a username must be addressed to this email: sispro_bodega@minsalud.gov.co. The researcher interested in accessing the data must request permission to enter the RUAF databases and the RIPS database, hosted by SISPRO. As the databases are not public and freely accessible; the researcher must explain the use that will be given to the data. After the request, the researcher will receive online training to access through the SQL Server Analysis Services cube in Excel, after the training, they will receive a username and password to use the databases. Figshare: Disease database. https://doi.org/10.6084/m9.figshare.20498970.v2 ( Gutierrez, 2022a). This project contains the following underlying data:
• Disease database.xlsx. (Contains the number of disease cases by department, age, and sex). Data are available under the terms of the Creative Commons Attribution 4.0 International license (CC-BY 4.0). Figshare: Mortality database. https://doi.org/10.6084/m9.figshare.20498964.v2 ( Gutierrez, 2022b). This project contains the following underlying data:
• Mortality database.xlsx. (Contains the number of deaths by cause, by department, age, and sex). Data are available under the terms of the Creative Commons Attribution 4.0 International license (CC-BY 4.0). Figshare: Departmental population projections by area, sex and age. https://doi.org/10.6084/m9.figshare.20499003.v3 ( Gutierrez, 2022c). This project contains the following underlying data:
• Departmental population projections by area, sex and age.xlsx. (Contains the number of inhabitants by department, age, and sex). Data are available under the terms of the Creative Commons Attribution 4.0 International license (CC-BY 4.0). Figshare: Table 2. Years of life lost due to premature death, Orinoquia (Colombia), 2017. https://doi.org/10.6084/m9.figshare.21231707.v2 ( Gutierrez, 2022d). This project contains the following underlying data:
-
Table 2. Years of life lost due to premature death, Orinoquia (Colombia), 2017 Data are available under the terms of the Creative Commons Attribution 4.0 International license (CC-BY 4.0). Figshare: Table 3. Table 3. Years lived with disability, Orinoquia (Colombia), 2017. https://doi.org/10.6084/m9.figshare.21231740.v1 ( Gutierrez, 2022e). This project contains the following underlying data:
-
Table 3. Years lived with disability, Orinoquia (Colombia), 2017. Data are available under the terms of the Creative Commons Attribution 4.0 International license (CC-BY 4.0). Figshare: Table 4. Disability-Adjusted Life Years, Orinoquia (Colombia), 2017. https://doi.org/10.6084/m9.figshare.21231752.v1 ( Gutierrez, 2022f). This project contains the following underlying data:
-
Table 4. Disability-Adjusted Life Years, Orinoquia (Colombia), 2017. Data are available under the terms of the Creative Commons Attribution 4.0 International license (CC-BY 4.0). Figshare: Sourcecode. https://doi.org/10.6084/m9.figshare.20522550.v2 ( Gutierrez, 2022g). This project contains the following underlying data:
• Sourcecode.pdf (Contains code for estimating years of life with disability (YLDs), by department, age, and sex) Data are available under the terms of the Creative Commons Attribution 4.0 International license (CC-BY 4.0). Figshare: STROBE checklist for ‘Burden of disease in Colombian Orinoquia, 2017’ https://doi.org/10.6084/m9.figshare.20499018.v1 ( Gutierrez, 2022h).

## Footnotes

^1^
A metric composed of per capita income, average educational level, and fertility rates to classify the development level of a country.
