# Supplementary material for: Burden of disease in Colombian Orinoquia Region, 2017
Source: F1000Res. 2023 Nov 20;11:1257. Originally published 2022 Nov 4. [Version 2] doi: 10.12688/f1000research.124503.2 (PMC11350327; doi:10.12688/f1000research.124503.2)
Supplement: Peer review history

# Reviewer report: Reviewer response for version 2

**DOI:** 10.5256/f1000research.158998.r273762

**Published:** 2024-05-08

**Authors:** Simin Mouodi

**Affiliations:** 1 Babol University of Medical Science, Babol, Iran

This research describes the burden of diseases in the Colombian region of Orinoquia.

 My recommendations have been listed as following: 

- The manuscript needs major grammatical editing.
- Please revise the "Keywords" according to main heading terms of the MeSH.
- Please revise the "Conclusion" and "Results" section of the "Abstract". Some findings mentioned in "Conclusion" section have not been reported in "Results".
- Why did the authors analyse the data related to year 2017? This year was seven years ago; and also, the impact of COVID-19 on DALYs had not been occurred.
- In "Introduction" section, these two sentences seem to be similar and can be merged:  

"there have been no burden of disease studies performed nor measurements of the non-fatal effects of diseases that can be used as a diagnosis of health status to guide health policies and programs taken".  AND

                 "Not having studies that measure the non-fatal effects of the disease causes an underestimation of the impact that these diseases have on the population, affecting decision-making processes".

  

- Please rearrange the subheadings of "Methods" section; for example: 1- Study design; 2- Setting; 3- Participants; 4- Variables; 5- Data sources/ measurement; 6- Bias; 7- Study size; 8- Statistical methods.
- Please write the word "DALY" in Figure 1, Figure 3 and Figure 4 with capital letters.

Is the work clearly and accurately presented and does it cite the current literature?

Partly

If applicable, is the statistical analysis and its interpretation appropriate?

Partly

Are all the source data underlying the results available to ensure full reproducibility?

Yes

Is the study design appropriate and is the work technically sound?

Partly

Are the conclusions drawn adequately supported by the results?

Partly

Are sufficient details of methods and analysis provided to allow replication by others?

Yes

Reviewer Expertise:

Medicine; Clinical Research

I confirm that I have read this submission and believe that I have an appropriate level of expertise to confirm that it is of an acceptable scientific standard, however I have significant reservations, as outlined above.

# Response

**Published:** 2024-05-08

**Authors:** OSCAR GUTIERREZ

**Affiliations:** Universidad de los Llanos, Colombia

The manuscript needs major grammatical editing.

**Response**:

 Dear reviewer: we carried out the edition under the editorial supervision of the magazine, with its team, below are our responses. We thank you in advance for your contributions.

 Please review "Keywords" in accordance with the terms of the main MeSH header.

**Response:**

 Dear reviewer: a review has been carried out and the keywords are consistent

 Please review the “Conclusion” and “Results” section of the “Summary”. Some findings mentioned in the "Conclusion" section have not been reported in "Results."

**Response:**

 Dear reviewer: The conclusions were verified, and if the results are reported, in the attached tables, thank you for your appreciation

 Why did the authors analyze data related to the year 2017? This year was seven years ago; and furthermore, the impact of COVID-19 on DALYs had not occurred.

**Response:**

 Dear reviewer: this research began in 2019, its protocol was approved in 2020, the most recent state data available at the beginning of the execution was from 2017, and to that, we must add the verification time, from the above it seems that the study took a date not recent.

 In the “Introduction” section, these two sentences appear to be similar and can be merged:

 "There have been no studies on the burden of disease or measurements of the non-fatal effects of diseases that can be used as a diagnosis of health status to guide adopted health policies and programs." AND

 "Not having studies that measure the non-fatal effects of the disease causes an underestimation of the impact that these diseases have on the population, affecting decision-making processes."

**Response:**

 Dear reviewer: the first sentence is the diagnostic expression about the absence of measurement in the departments, the second sentence is required as an introduction to present the effects of the absence of this type of studies. We merged them, but it resulted in a somewhat long paragraph, we considered with your approval to keep the two sentences in separate paragraphs.

 Please rearrange the subheadings of the "Methods" section; for example: 1- Study design; 2- Setting; 3- Participants; 4- Variables; 5- Data/measurement sources; 6- Bias; 7- Size of the study; 8- Statistical methods.

 Write the word "DALY" in Figure 1, Figure 3 and Figure 4 with capital letters.

**Response:**

 Dear Reviewer: We agree with the review; however, we are under the journal's editing guidelines.

# Reviewer report: Reviewer response for version 2

**DOI:** 10.5256/f1000research.158998.r224070

**Published:** 2023-11-28

**Authors:** Ababi Zergaw

**Affiliations:** 1 School of Public Health, Addis Ababa University, Addis Ababa, Addis Ababa, Ethiopia

I found comments provided in my review are well addressed and feel the responses given by the authors are very interesting particularly their justification for the methodology that they have used is very much appreciated.

Is the work clearly and accurately presented and does it cite the current literature?

Partly

If applicable, is the statistical analysis and its interpretation appropriate?

I cannot comment. A qualified statistician is required.

Are all the source data underlying the results available to ensure full reproducibility?

Yes

Is the study design appropriate and is the work technically sound?

Partly

Are the conclusions drawn adequately supported by the results?

Yes

Are sufficient details of methods and analysis provided to allow replication by others?

Yes

Reviewer Expertise:

NA

I confirm that I have read this submission and believe that I have an appropriate level of expertise to confirm that it is of an acceptable scientific standard.

# Reviewer report: Reviewer response for version 1

**DOI:** 10.5256/f1000research.136706.r219002

**Published:** 2023-11-14

**Authors:** Ababi Zergaw

**Affiliations:** 1 School of Public Health, Addis Ababa University, Addis Ababa, Addis Ababa, Ethiopia

As argued and the given rationale for the study of the “Burden of disease in Colombian Orinoquia, 2017” it crucial to describe level of disease burden for better policy and health system strengthening. The manuscript has used established methods of disease measurement to describe burden in Orinoquia, Colombia. Additionally the following comments will improve the research write-up further.

**General comment**:

1. Better to refine the title of the study as “Burden of disease in Colombian Orinoquia Region, 2017.”
2. Better to simplify some complex sections of the paper (see comments below) and avoid inconsistency in some wordings (DALYs vs DALY, synthetic indicator vs. composite indicator, territory vs territories, etc) in the text and needs minor editorial work for style (eg. starting a sentence or paragraph by figures).
3. The manuscript needs to put some descriptions in their appropriate sections particularly with the result and discussion part (some statements in the result section are better taken to discussion section and vice versa).
4. Almost seven years has elapsed since the study period 2017, so, it might be less impactful research result, which may need update.

**Abstract Section**:

1. In the conclusion section of the abstract, the statements from line 2 to 7 are better kept in result section and provide conclusion then after.

**The Introduction section**:

1. Much of the introduction sections states more about measurement tool DALYs rather than about disease burden issues as expected for a reader. So, it is good to minimize that.

**Methods section**:

1. In the manuscript, it is not clear what synthetic DALY means and how a projected age can be used as a measure for life expectancy in relation to the study setting. I think such use of future age projection in estimating YLL is questionable, for instance, because so many factors may come to play for the achievability of the projected age 92, e.g. health service access, quality, life style, education, fertility, social and economic factors, etc.
2. If I understand correctly what synthetic DALY means, this study has tried to use a mixed method from GHE and GBD studies (estimating YLL using SLE recommended by WHO which is 90 years and estimating YLD according to GBD 2019 disability weights for each health). So, in that case the study has developed a new method of measuring disease burden which needs explanation and ensuring its validity. Furthermore, it looks that combining databases needs to be checked for consistency and triangulation and make clear how these are done.
3. In the text description of this section, I found it difficult how primary diagnoses for cause of death were used (how about comorbidities, does adjustment made as recommended by simplified DALYs). Mix of methodologies like GHE, GBD Study, analytic tool like python 3.0 and other methods are combined bias control was applied on an already collected data kept at a database, It would be better if some more explanations are provided for sake of clarity.
4. As stated in #2 and 3, the statistical analysis part looks a bit complex.

**Result Section**:

1. In this section I found it difficult to examine how a result published in 2019 GBD study is used in this present study to support the findings of this 2017 Burden of disease study in the region.
2. Also in this section, it is good to support text descriptions with tables for a better understanding of specific figures. In the section, some tables e. g. Tables 2, 3 and 4 are not shown in the manuscript but descriptions are provided. Additionally, in figure 1, the age grouping does not look appropriate and in figure 2 these age groupings are merged (0-4 in fig. 1 vs 0-9 in fig. 2).

**Discussion section**:

1. In line with the age descriptions cited above, it looks difficult to make comparisons with other GBD and international studies with standard age categorizations.
2. As the study results are not well focused, it is difficult to identify major health problems for intervention selection or recommendation of appropriate action with priority and resource allocation. Even though it may not be appropriate to have a separate recommendation section, it may be nice to give some recommendations at to the choice of interventions and future research and answer the so what question.

Is the work clearly and accurately presented and does it cite the current literature?

Partly

If applicable, is the statistical analysis and its interpretation appropriate?

I cannot comment. A qualified statistician is required.

Are all the source data underlying the results available to ensure full reproducibility?

Yes

Is the study design appropriate and is the work technically sound?

Partly

Are the conclusions drawn adequately supported by the results?

Yes

Are sufficient details of methods and analysis provided to allow replication by others?

Yes

Reviewer Expertise:

NA

I confirm that I have read this submission and believe that I have an appropriate level of expertise to confirm that it is of an acceptable scientific standard, however I have significant reservations, as outlined above.

# Response

**Published:** 2023-11-15

**Authors:** OSCAR GUTIERREZ

**Affiliations:** Universidad de los Llanos, Colombia

As argued and the given rationale for the study of the “Burden of disease in Colombian Orinoquia, 2017” it crucial to describe level of disease burden for better policy and health system strengthening. The manuscript has used established methods of disease measurement to describe burden in Orinoquia, Colombia. Additionally the following comments will improve the research write-up further.

**General comment**:

1. Better to refine the title of the study as “Burden of disease in Colombian Orinoquia Region, 2017.”

**Response:** Ok, title adjusts

1. Better to simplify some complex sections of the paper (see comments below) and avoid inconsistency in some wordings (DALYs vs DALY, synthetic indicator vs. composite indicator, territory vs territories, etc) in the text and needs minor editorial work for style (eg. starting a sentence or paragraph by figures).

**Response:** OK, Adjusted, the paragraph that began with figures is edited, the writing of DALYs was homogenized, the mention of synthetic when referring to the DALYs was deleted, regarding the use of the word territory and territories, in the singular we use it to refer to the region , and the plural to the departments that are part of the region. 

1. The manuscript needs to put some descriptions in their appropriate sections particularly with the result and discussion part (some statements in the result section are better taken to discussion section and vice versa).

**Response**: Dear reviewer, we do not agree with this suggestion.

1. Almost seven years has elapsed since the study period 2017, so, it might be less impactful research result, which may need update.

Response: Regarding the year, this occurred due to the availability of the data, at the beginning of the work in 2019, however, there are no important changes in the epidemiological profile of the region, added to the above, as it is the first investigation estimating the burden of the disease, the results are useful as a baseline of the burden for the region and its departments.

**Abstract Section**:

1. In the conclusion section of the abstract, the statements from line 2 to 7 are better kept in result section and provide conclusion then after.

**Response**: Dear author, we consider these lines pertinent as conclusions

**The Introduction section**:

1. Much of the introduction sections states more about measurement tool DALYs rather than about disease burden issues as expected for a reader. So, it is good to minimize that.

**Response:** Dear reviewer, we consider it pertinent due to the need to present the methodology that is little used in Colombia.

**Methods section**:

1. In the manuscript, it is not clear what synthetic DALY means and how a projected age can be used as a measure for life expectancy in relation to the study setting. I think such use of future age projection in estimating YLL is questionable, for instance, because so many factors may come to play for the achievability of the projected age 92, e.g. health service access, quality, life style, education, fertility, social and economic factors, etc.

**Response:** The use of 92 years as a standard life expectancy obeys the theoretical postulate of the YLL, which defines that the Standard Life Expectancy must represent the demographic reality.

 As authors we define: The GBD methodology has modified the EVE; From 1993 to 2008, according to sex, 80 years for men and 82.5 for women were used with discount rates; Since 2010, for ethical considerations, the GBD methodology eliminates differences by sex, discount rates and used the 86 years without differences by sex and without a discount rate.

 As of 2013, the Global Health Estimation (GHE) methodology of the World Health Organization (WHO), uses a version of the GBD, calculates the YLD the same, but for the YLL it uses a Standard life expectancy (SLE) of 92 years old; This is a future projection for the year 2050 by the United Nations Population Division. The SLE, used in the GHE methodology, derives in new loss functions for the calculation of the YLLe; This represents the maximum life expectancy of a person in good health, not exposed to serious risks or injuries, and receiving adequate health services.

 Therefore, in consideration of the theoretical postulate of defining an (SLE), which covers all ages presented in the territory, and does not cause underestimation in YLLs.

 Now, when the guide was revised: “WHO methods and data sources for global burden of disease estimates 2000-2011. Geneva: Department of Health Statistics and Information Systems: Global Health Estimates Technical Paper WHO/HIS/HSI/GHE/” it was found that: when comparing the weighting factors obtained from the different SLE (GBD vs. GHE), from the GHE methodology , the 2010 GBD methodology, but an SLE of 92 years (GHE) vs. 86 years (GBD), the weighting factors in the 2010 GBD are lower by 6.43% in neonates and lower by 33.5% in those over 85 years of age.

1. If I understand correctly what synthetic DALY means, this study has tried to use a mixed method from GHE and GBD studies (estimating YLL using SLE recommended by WHO which is 90 years and estimating YLD according to GBD 2019 disability weights for each health). So, in that case the study has developed a new method of measuring disease burden which needs explanation and ensuring its validity. Furthermore, it looks that combining databases needs to be checked for consistency and triangulation and make clear how these are done.

**Response**: Dear reviewer, it is not a new method, it is the method used by the WHO in its GHE, which as presented and developed by the WHO in “Methods and data sources for global burden of disease estimates 2000-2011. Geneva: Department of Health Statistics and Information Systems: Global Health Estimates Technical Paper, the same GBD method, but with the SLE variation. The updated disability weights were used for the calculations of the YLD in GBD in 2019, as indicated by the GHE, and the YLL according to the GBD but with SLE of the GHE.

 The use of the term synthetic in the document is more of a semantic inclination of ours, we have already eliminated it from the manuscript, using the term synthetic comes from a mention made by the authors to define its metric nature, as it is called in its construction and to differentiate it from the simple indicators, in the presentation of the research protocol to juries and the academic community, therefore, following their recommendation, the synthetic term was already removed from the manuscript.

1. In the text description of this section, I found it difficult how primary diagnoses for cause of death were used (how about comorbidities, does adjustment made as recommended by simplified DALYs). Mix of methodologies like GHE, GBD Study, analytic tool like python 3.0 and other methods are combined bias control was applied on an already collected data kept at a database, It would be better if some more explanations are provided for sake of clarity.

**Response:** We reiterate, we did not combine methodology, GHE was applied.

 Regarding the cause of death, which can be affected by the competing risk of mortality, this cannot be controlled when a secondary source is used, as is the case of this research, but the recommendation for mortality studies in populations was followed when the source They are vital statistics. The basic cause of death declared in the mortality bases of the National Department of Statistics (DANE) of Colombia was used. The underlying cause is defined in the Colombian Vital Statistics System under the rules and guidelines for the coding of mortality and morbidity, adopted by the World Health Assembly in relation to the selection of a single cause or condition for tabulation. routine death certificates and morbidity records.

 The intervention of biases is found in the bias control section.

 The other limitations, such as adjustments for comorbidity that affect the estimation and control for other biases, are recorded in limitations.

 Yes, we found an error thanks to your review, in the limitations we declared problems in the estimation of YLDs due to comorbidity adjustment, but due to a writing error, we wrote YLL, we made the change in the limitations statement.

1. As stated in #2 and 3, the statistical analysis part looks a bit complex.

**Result Section**:

1. In this section I found it difficult to examine how a result published in 2019 GBD study is used in this present study to support the findings of this 2017 Burden of disease study in the region.

**Response:** Dear reviewer, we are clear that we start from a difference in the SLE used to calculate the YLL of this research and the IHME, however, when using the results of the IHME studies, we do not compare the value of the estimate, but rather the order or classification achieved by each disease. All the YLL estimated in the Orinoquia used the same SLE, allowing their classification with the same magnitude, the same as the YLL of the IHME in their studies. Therefore, we compare only their location or classification, to note the order of importance of the disease. We do the same comparison logic with some studies carried out in Colombia with the 1993 GBD methodology, we only compare the order or classification of the events. Finally, we also report this situation in the limitations section.

1. Also in this section, it is good to support text descriptions with tables for a better understanding of specific figures. In the section, some tables e. g. Tables 2, 3 and 4 are not shown in the manuscript but descriptions are provided. Additionally, in figure 1, the age grouping does not look appropriate and in figure 2 these age groupings are merged (0-4 in fig. 1 vs 0-9 in fig. 2).

**Reponses:** Dear reviewer, the tables mentioned, but not present in the text, due to their large size, should have been published online, found in links in the article.

 His observations on figures 1 and 2 were adequate, therefore these figures were edited and transformed according to his request.

**Discussion section**:

1. In line with the age descriptions cited above, it looks difficult to make comparisons with other GBD and international studies with standard age categorizations.

**Response:** Dear reviewer, we are clear that we start from a difference in the SLE used to calculate the YLL of this research and the IHME, however, when using the results of the IHME studies, we do not compare the value of the estimate, but rather the order or classification achieved by each disease. All the YLL estimated in the Orinoquia used the same SLE, allowing their classification with the same magnitude, the same as the YLL of the IHME in their studies. Therefore, we compare only their location or classification, to note the order of importance of the disease. We do the same comparison logic with some studies carried out in Colombia with the 1993 GBD methodology, we only compare the order or classification of the events. Finally, we also report this situation in the limitations section.

1. As the study results are not well focused, it is difficult to identify major health problems for intervention selection or recommendation of appropriate action with priority and resource allocation. Even though it may not be appropriate to have a separate recommendation section, it may be nice to give some recommendations at to the choice of interventions and future research and answer the so what question.

**Response:** Dear reviewer; there is no recommendations section

# Reviewer report: Reviewer response for version 1

**DOI:** 10.5256/f1000research.136706.r157450

**Published:** 2023-01-04

**Authors:** Pablo Enrique Chaparro Narváez

**Affiliations:** 1 Instituto Nacional de Salud, Bogotá, Colombia

This is an interesting paper about the burden of disease in the Colombian Orinoquia in 2017. The data is interesting. However, there are a few suggestions that should to consider, such as follows:

**Abstract**:

- To move the aim to the end of the background.
- The conclusion must be synthetic.
- To include recommendation(s).

**Keywords**:

- To review in MeSH: "health profile".

**Introduction**:

- The objective should include the time frame of analysis.

**Methods**:

- What is the definition of "miscoded mortality cases"?
- The study was carried out in 2017. Then the population projections used were those corresponding to that year and not from 2005 to 2017.
- In Colombia, the underlying cause of death is not determined by the treating physician. The underlying cause of death is selected according to the norms established by the international statistical classification of diseases and related health problems, tenth revision.
- How was the miscoding in morbidity determined?
- To include calculations of:
  - Frequencies.
  - Crude rate.
  - Standardized rates and the reference population used.
  - The process used for the distribution of miscoded cases.
  - The adjustment with the underreporting reported by Hill's method.

**Discussion**:

- To specify which countries are referred to by "Latin America and the Caribbean" and "other countries of the world". It is a repetitive aspect in this section.
- Why was the WHO standard life expectancy used? It is about 10 years above that of Colombia.

- The following aspects have not been discussed:
  - The differences in the results between the departments of the Orinoquia.
  - The underreporting of mortality obtained by the authors compared to that reported by other studies.
  - The underreporting of morbidity compared to that reported by other studies.
  - The disability weights obtained from the IHME.
- Highlight recommendations.

**Data availability**:

 Mortality information is public and freely accessible. It is available in DANE, Estadísticas por tema, Sociedad, Demografía y población, Nacimientos y Defunciones, Defunciones, Microdatos anonimizados https://www.dane.gov.co/index.php/estadisticas-por-tema/demografia-y-poblacion/nacimientos-y-defunciones/estadisticas-vitales-nacimientos-y-defunciones-historicos or http://microdatos.dane.gov.co/index.php/catalog/MICRODATOS/about_collection/22/5

Is the work clearly and accurately presented and does it cite the current literature?

Yes

If applicable, is the statistical analysis and its interpretation appropriate?

Yes

Are all the source data underlying the results available to ensure full reproducibility?

Yes

Is the study design appropriate and is the work technically sound?

Yes

Are the conclusions drawn adequately supported by the results?

Yes

Are sufficient details of methods and analysis provided to allow replication by others?

No

Reviewer Expertise:

Public health, epidemiology

I confirm that I have read this submission and believe that I have an appropriate level of expertise to confirm that it is of an acceptable scientific standard, however I have significant reservations, as outlined above.

# Response

**Published:** 2023-01-06

**Authors:** OSCAR GUTIERREZ

**Affiliations:** Universidad de los Llanos, Colombia

**Abstract**:

- To move the aim to the end of the background.
- The conclusion must be synthetic.
- To include recommendation(s).

**Response:** The abstract was made according to the ARTICLE GUIDELINES of F1000, the requests contravene them, therefore the request to include recommendations in the abstract cannot be accepted.

**Keywords**:

- To review in MeSH: "health profile".

**Response**: The review of the keyword health profile was carried out, it has a unique identifier DDCS016859, and it is consistent intending to the research.

**Introduction**:

- The objective should include the time frame of analysis.

**Response**: The authors make the adjustment requested by the reviewer.

**Methods**:

- What is the definition of "miscoded mortality cases"?

**Response**: miscoded mortality cases refers to the classification that the GBD methodology makes of some codes (garbage codes).

- The study was carried out in 2017. Then the population projections used were those corresponding to that year and not from 2005 to 2017.

**Response**: Yes Doctor, the year used is 2017, adjustment made by the authors.

- In Colombia, the underlying cause of death is not determined by the treating physician. The underlying cause of death is selected according to the norms established by the international statistical classification of diseases and related health problems, tenth revision.

**Response**: Yes, doctor, but the person responsible for filling out the certificate and selecting the causes applying the established rules for classification is the attending physician or legal medicine, to which reference is made regarding the source of the data (death certificates).

 This is what is mentioned in the article:

 “These reports are made by the treating physician or the forensic doctor through electronic platforms, with the medical reports consolidated in SISPRO, after quality control”.

 Adjustment is made for clarity by the reviewer's request, thus remaining:

 “These reports are made by the treating physician or the forensic doctor through electronic platforms, with the medical reports consolidated in SISPRO, after quality control by DANE”.

- How was the miscoding in morbidity determined?

**Response**: It was not possible to determine or adjust, in the metric used there is only miscoding for mortality

- To include calculations of:
  - Frequencies.

**Response**: The frequencies are those reported in the databases used, grouped according to the classification of the metric. This is how it is described in the methodology

-
  - Crude rate. OK
  - Standardized rates and the reference population used. **OK**

**Response**: Adjusted per request:

 Including in the footer of the table: the reference population used for the standardized rates, mentioning the numerator and denominator of the population

  

-
  - The process used for the distribution of miscoded cases.

**Response**: The GBD methodology defines the garbage codes (these were identified for the Orinoquia records), the GBD methodology also defines their redistribution in the corresponding codes, this distribution was made by proportional apportionment according to sex and age of the cases of garbage codes. did in the codes defined by the GBD.

  

-
  - The adjustment with the underreporting reported by Hill's method.

**Response**: The percentage (%) of underreporting was applied to expand the number of cases, proportionally (apportionment) by age, sex and event, bringing the Orinoquia mortality databases to a theoretical completeness.

**Discussion**:

- To specify which countries are referred to by "Latin America and the Caribbean" and "other countries of the world". It is a repetitive aspect in this section.

**Response**: The list of countries is too extensive to mention them all in the lines that are cited in the article, for this reason, they were grouped into those categories, they were grouped for purposes of order and style.

- Why was the WHO standard life expectancy used? It is about 10 years above that of Colombia.

**Response:** The main theoretical assumption of the YLL is a life expectancy, a standard that allows the comparability of the territories and/or time, which at the same time does not generate underestimation by leaving out subjects that exceed the life expectancy of a territory. Under this principle, the WHO sets life expectancy at 92 years for global disease burden estimation studies. We assume the same because it is the theoretical assumption of the metric for the comparability of results and to avoid underestimation of YLL and DALYs.

- The following aspects have not been discussed:
  - The differences in the results between the departments of the Orinoquia.

**Response:** You are right, differences between departments are not mentioned, because the unit of territorial analysis of this article was the Orinoquia region.

-
  - The underreporting of mortality obtained by the authors compared to that reported by other studies.

**Response:** There are no studies on underreporting for the territory in the period studied, with methods based on two censuses. In the article referenced by the authors "Completion of mortality reports in the vital statistics system in the Colombian Orinoquía, 2017" used as a reference of the underreporting of the region, if you can find a discussion regarding the underreporting for the region through different metrics.

 The mention of underreporting was a step to demonstrate the adjustment made by the authors, as necessary to control the underestimation of the YLL of the DALYs, but it is not the objective of this article.

-
  - The underreporting of morbidity compared to that reported by other studies.

**Response:** This research could not estimate this underreporting, it is mentioned as a limitation in the limitations section.

-
  - The disability weights obtained from the IHME.

**Response:** These weights are standardized, defined in other investigations that had the purpose of creating the weights, these are used as input in the GBD and GHE methodology, these weights are not the subject of results or discussion of this article, they are referenced by the authors in this article.

- Highlight recommendations.

**Data availability**:

 Mortality information is public and freely accessible. It is available in DANE, Estadísticas por tema, Sociedad, Demografía y población, Nacimientos y Defunciones, Defunciones, Microdatos anonimizados https://www.dane.gov.co/index.php/estadisticas-por-tema/demografia-y-poblacion/nacimientos-y-defunciones/estadisticas-vitales-nacimientos-y-defunciones-historicos or http://microdatos.dane.gov.co/index.php/catalog/MICRODATOS/about_collection/22/5

**Response:** Identification of each case is required by cause of death, according to the CIE (to achieve the GBD classification), year, sex, age and territory.

 The present investigation used the following data, which also come from a public source of the Colombian state produced by DANE stored in SISPRO. Available in the article:

 Figshare: Mortality database. https://doi.org/10.6084/m9.figshare.20498964.v2 (Gutierrez, 2022b).

 This project contains the following underlying data:

 • Mortality database.xlsx. (Contains the number of deaths by cause, by department, age, and sex).

 Data are available under the terms of the Creative Commons Attribution 4.0 International license (CC-BY 4.0).
